# Supplementary material for: Comparison of the Antibiotic Resistance of Escherichia coli Populations from Water and Biofilm in River Environments
Source: Pathogens. 2024 Feb 13;13(2):171. doi: 10.3390/pathogens13020171 (PMC10891912; doi:10.3390/pathogens13020171)
Supplement: Supplementary file 1 [file pathogens-13-00171-s001.zip › Supplementary Table S5.pdf]

Supplementary Table S5. Antibiotic resistances of *E. coli* Drava river isolates from upstream and downstream of the WWTP in comparison between water and sediment. The proportions, and in parentheses, the number of isolates resistant to each antibiotic and for all classes of antimicrobial resistance are given with the corresponding p-values of the statistical tests. Isolates were classified as wildtype when showing no resistance to the tested antibiotics. Isolates with resistance to one or two classes of the tested antibiotics were classified as resistant. Resistance to three or more classes of the tested antibiotics was classified as multi-resistant. P-values < 0.05 were considered as statistically significant. P-values with more than four decimal places containing a value of nine were rounded to one. us – upstream of the WWTP; ds – downstream of the WWTP.

	**Drava water us** (90 isolates)	**Drava sediment us** (33 isolates)	**p-value**	**Drava water ds** (105 isolates)	**Drava sediment ds** (34 isolates)	**p-value**
**β-Lactams**						
Ampicillin	13.33 % (12)	18.18 % (6)	0.57	16.19 % (17)	11.76 % (4)	0.78
Amoxicillin/ clavulanic acid	12.22 % (11)	18.18 % (6)	0.39	10.48 % (11)	8.82 % (3)	1
Cefalexin	2.22 % (2)	0 % (0)	1	2.86 % (3)	0 % (0)	1
Cefuroxime	2.22 % (2)	0 % (0)	1	1.9 % (2)	0 % (0)	1
Cefoxitin	1.11 % (1)	0 % (0)	1	0.95 % (1)	0 % (0)	1
Cefotaxime	2.22 % (2)	0 % (0)	1	0.95 % (1)	0 % (0)	1
Piperacillin/ Tazobactam	0 % (0)	0 % (0)	1	0 % (0)	5.88 % (2)	0.06
Ceftazidime	2.22 % (2)	0 % (0)	1	0 % (0)	0 % (0)	1
Cefepime	2.22 % (2)	0 % (0)	1	0.95 % (1)	0 % (0)	1
Imipenem	0 % (0)	0 % (0)	1	0 % (0)	0 % (0)	1
Meropenem	0 % (0)	0 % (0)	1	0 % (0)	0 % (0)	1
**Quinolones**						
Moxifloxacin	2.22 % (2)	6.06 % (2)	0.29	8.57 % (9)	0 % (0)	0.11
Ciprofloxacin	3.33 % (3)	6.06 % (2)	0.61	8.57 % (9)	11.76 % (4)	0.52
Nalidixic acid	8.89 % (8)	12.12 % (4)	0.73	9.52 % (10)	0 % (0)	0.12
**Tetracyclines**						
Tetracycline	4.44 % (4)	3.03 % (1)	1	8.57 % (9)	0 % (0)	0.11
Tigecycline	0 % (0)	0 % (0)	1	0 % (0)	0 % (0)	1
**Aminoglycosides**						
Gentamicin	1.11 % (1)	3.03 % (1)	0.47	0 % (0)	2.94 % (1)	0.24
Amikacin	0 % (0)	0 % (0)	1	0.95 % (1)	0 % (0)	1
**Antifolate**						
Trimethoprim/ sulfamethoxazole	8.89 % (8)	12.12 % (4)	0.73	10.48 % (11)	0 % (0)	0.07
**Polymyxins**						
Colistin	0 % (0)	0 % (0)	1	0 % (0)	0 % (0)	1
**Chloramphenicols**						
Chloramphenicol	1.11 % (1)	6.06 % (2)	0.18	2.86 % (3)	0 % (0)	1
