# Supplementary material for: Comparison of the Antibiotic Resistance of Escherichia coli Populations from Water and Biofilm in River Environments
Source: Pathogens. 2024 Feb 13;13(2):171. doi: 10.3390/pathogens13020171 (PMC10891912; doi:10.3390/pathogens13020171)
Supplement: Supplementary file 1 [file pathogens-13-00171-s001.zip › Supplementary Table S7.pdf]

Supplementary Table S7. Primers used for PCR.

Gene Families	Primer	Sequence 5′→3′	Reference
bla$_{CTX-M-1-group}$	f	TCTTCCAGAATAAGGAATCCC	[22]
	r	CCGTTTCCGCTATTACAAAC	
bla$_{CTX-M-2-group}$	f	ATGATGACTCAGAGCATTCG	[24]
	r	TGGGTTACGATTTTCGCCGC	
bla$_{CTX-M-9-group}$	f	ATGGTGACAAAGAGAGTGCA	[23]
	r	CCCTTCGGCGATGATTCTC	
bla$_{SHV}$	f	TGGTTATGCGTTATATTCGCC	[25]
	r	GGTTAGCGTTGCCAGTGCT	
bla$_{TEM}$	f	TCCGCTCATGAGACAATAACC	[22]
	r	TTGGTCTGACAGTTACCAATGC	
Bla$_{KPC}$	f	CAGCTCATTCAAGGGCTTTC	[26]
	r	AGTCATTTGCCGTGCCATAC	
